# Can neurologic music therapy make the difference when using immersive virtual reality in Parkinson disease motor training? Promising findings from a secondary analysis

**DOI:** 10.3389/fresc.2026.1707528

**Published:** 2026-02-26

**Authors:** Paolo De Pasquale, Mirjam Bonanno, Antonino Lombardo Facciale, Maria Grazia Maggio, Michael H. Thaut, Corene Hurt, Angelo Quartarone, Federica Impellizzeri, Rocco Salvatore Calabrò

**Affiliations:** 1 IRCCS Centro Neurolesi Bonino-Pulejo, Messina, Italy; 2 Music and Health Science Research Collaboratory, Faculty of Music, Faculty of Medicine, Institute of Medical Sciences and Rehabilitation Science Institute, University of Toronto, Toronto, ON, Canada; 3 Department of Biomedical, Dental Sciences and Morphological and Functional Images, University of Messina, Messina, Italy

**Keywords:** biomechanics of gait, CAREN, gait analysis, gait and balance, neurologic music therapy, neurorehabilitation, Parkinson disease, virtual reality

## Abstract

**Background and purpose:**

This study presents a secondary analysis of a quasi-randomized clinical trial exploring the effects of immersive virtual reality (VR) rehabilitation alone versus in combination with Neurologic Music Therapy (NMT) in individuals with Parkinson's disease (PD).

**Methods:**

Twenty participants with idiopathic Parkinson's disease were allocated to two groups using a quasi-randomized procedure. One group received immersive VR-based gait training using the CAREN system (CAREN group), while the other received the same training protocol with the addition of NMT sensory-motor techniques delivered by a certified NMT therapist (CAREN-M group). Participants attended 12 rehabilitation sessions (three sessions per week over four weeks), each lasting approximately 45 min. Both groups underwent the same number, duration, and intensity of rehabilitation sessions. Clinical outcomes (BBS, Tinetti Scale, Barthel Index, 10-meter walking test, Timed-Up-Go) and biomechanical gait parameters (kinematic, kinetic, and electromyography data) were assessed for pre- and post-treatment.

**Results:**

Both interventions led to significant improvements in motor function, but group-specific patterns emerged. The CAREN group showed greater gains in spatial parameters, such as gait speed and stride length, alongside increased muscle activation and improved joint kinematics. The CAREN-M group exhibited superior improvements in temporal parameters (e.g., cycle and swing duration), trunk stability, and postural control, suggesting that rhythmic auditory stimulation (RAS) may enhance motor timing and coordination. Between-group comparisons showed a greater reduction in Time Up and Go (TUG) times in the CAREN-M group.

**Discussion and conclusion:**

These findings support the hypothesis that combining immersive VR (e.g., CAREN system) with NMT offers additive benefits by targeting distinct components of motor control through multisensory stimulation. The integration of rhythmic auditory cues into immersive VR environments can represent a promising direction for neurorehabilitation in PD, with the potential to improve functional mobility and gait quality.

**Trial Registration Number:**

NCT07066137.

## 1 Introduction

Parkinson disease (PD) is one of the most prevalent neurodegenerative disorders worldwide, characterized by a complex constellation of motor and non-motor symptoms (1). It is worth noting that patients with PD experience biomechanical gait alterations, even in the primary stages of disease (2), including reduced walking speed and stride length, increased cadence, prolonged double-limb support, and disrupted gait rhythm (3). In this context, conventional rehabilitation approaches for PD have long been designed to target specific motor deficits, enhance mobility, and functional independence in PD patients (4). Although traditional motor rehabilitation approaches (e.g., step training over obstacles and/or the Bobath approach), can address some PD-related deficits, they often lack the standardization of repetitions and multisensory stimulation, which are required to fully engage patients or target the complexity of gait and balance impairments (5). In recent years, the landscape of PD rehabilitation has been enriched by advanced technologies that promote motor recovery and functional enhancement through principles of neuroplasticity and task-oriented motor learning. Among these, immersive virtual reality (VR) combined with treadmill-based training has emerged as a promising tool to promote functional recovery (6, 7). Treadmill-based rehabilitation enables dynamic, adaptable and task-specific gait training protocols tailored to address specific impairments commonly observed in PD (8, 9), while immersive VR environments offer interactive, ecologically valid settings where patients can engage in complex motor tasks with multisensory feedback and heightened motivation (10–13). According to Brandín-De la Cruz et al. (14), immersive VR treadmill training is feasible and well accepted by individuals with PD and may improve performance on the 10-meters walking test after treatment. Similar findings were reported by de Melo et al. (15), who observed an increase in 6-min walking distance (approximately from 52 to 87 m) and faster gait following a 4-week program (three sessions per week) of treadmill training with immersive virtual reality compared with a control intervention. Immersive VR systems, such as Computer Assisted Rehabilitation Environment (CAREN, Motek, Netherlands), offer a major advantage by delivering simultaneous auditory, visual, and proprioceptive cues, promoting higher engagement and facilitating neuroplastic changes (16). The CAREN system is an advanced rehabilitation platform that integrates a split-belt treadmill mounted on a six-degrees-of-freedom motion base with a large immersive VR screen and a Vicon motion-capture system. During training, the patient walks or stands on the treadmill/platforms while the VR environment provides task-specific visual stimuli (e.g., obstacles, path changes, balance task, dual-task cues). Importantly, the platform can deliver controlled translations/rotations (i.e., perturbations) synchronized with the virtual scene, thereby increasing sensorimotor coupling and enabling ecologically valid gait challenges. Compared to other treadmill-based VR systems, which typically rely on a stationary treadmill, limited immersion (e.g., standard monitors or simpler visual feedback), and less integrated motion tracking, the CAREN allows programmable multi-directional perturbations, supporting a real-time integration of cognitive and motor demands. Despite these advantages, the CAREN system does not provide rhythmic auditory stimulation (RAS). RAS is a specific technique of Neurologic Music Therapy (NMT) that has proved its effectiveness in PD patients (17, 18) since it can compensate for the loss of automatic and rhythmic movements (19–21).

In a previous RCT by our research group, RAS, in addition to treadmill-based gait training in PD patients, reshaped sensorimotor rhythms and fronto-centroparietal/temporal connectivity. RAS has demonstrated efficacy in improving gait parameters, enhancing timing, and stabilizing movement patterns in individuals with PD (20, 22). These neurophysiological findings align with the broader framework suggesting that musical rhythms can entrain movement patterns and provide a continuous temporal reference that helps regulate movement timing and pace in movement disorders (23, 24). When applied within innovative settings (such as musical treadmills or immersive VR systems), musical stimuli can provide a structured, engaging, and adaptive framework for rehabilitation. This combination not only enhances movement coordination and gait stability but also increases patient engagement and adherence (21, 25). The integration of RAS into immersive VR environments represents a novel and underexplored synergy, with the potential to optimize motor learning through structured rhythmic feedback.

The present study represents a secondary motor analysis of a previously published quasi-randomized clinical trial that investigated the effects of combining NMT with immersive VR on executive and cognitive functions in individuals with PD (16). The objective of the present work is therefore to explore whether the integration of immersive VR and NMT techniques may lead to greater improvements in motor performance compared to immersive VR alone.

## 2 Methods

### 2.1 Study design

This secondary analysis focuses specifically on motor outcomes derived from a previous experimental protocol approved by the local Ethics Committee (IRCCS-ME-23/2022) and conducted in accordance with the Declaration of Helsinki. All participants provided written informed consent prior to enrolment. The study is part of a broader umbrella protocol registered on ClinicalTrials.gov (NCT07066137).

### 2.2 Participants

Twenty individuals diagnosed with idiopathic PD were included in this secondary analysis. Participants were allocated to the two intervention groups using a quasi-randomized assignment procedure based on order of enrolment. Specifically, participants were alternately assigned to the CAREN or CAREN-M group as they entered the study, ensuring balanced group sizes. No stratification or block randomization was applied. Given the exploratory nature of the parent trial, this allocation strategy was chosen to ensure feasibility while minimizing group imbalance. Baseline demographic and clinical characteristics did not differ significantly between groups, reducing the risk of major selection bias. The first group underwent motor rehabilitation using an immersive virtual reality system (CAREN group, *n* = 10), while the second group received the same intervention with the addition of NMT motor techniques (CAREN-M group, *n* = 10).

In the parent trial, eligibility criteria included a confirmed diagnosis of PD according to the Movement Disorder Society (MDS) clinical diagnostic criteria (26), an age range of 40–80 years, and at least five years of formal education. Although the primary report focused on individuals with mild-to-moderate disease severity (Hoehn & Yahr ≤ 2.5), the trial database also included participants with higher Hoehn & Yahr stages who were excluded from the primary analysis. In the present secondary analysis, we included all available participants with complete motor outcome data, without restricting inclusion to a specific Hoehn & Yahr stage (Table 1), provided that they were able to walk independently (with or without an assistive device). No participants were drawn from other studies or trials.

**Table 1:** Demographic and clinical characteristics of the sample.

Characteristics	All	CAREN M	CAREN	*p*-Value	Test
Patients	20 (100%)	10 (50%)	10 (50%)		
Age	65.2 ± 8.23	62.2 ± 6.84	68.2 ± 8.73	0.1	t-test
Education	11.55 ± 5.63	13.4 ± 7.11	9.7 ± 2.98	0.34	U
Gender					
Male	17 (85%)	8 (80%)	9 (90%)	1	Fisher
Female	3 (15%)	2 (20%)	1 (10%)	1	Fisher
Hoehn &Yahr					
Stage 1	1 (5)	0 (0)	1 (10)	1	Fisher
Stage 1.5	2 (10)	2 (20)	0 (0)	0.47	Fisher
Stage 2	7 (35)	3 (30)	4 (40)	1	Fisher
Stage 3	6 (30)	4 (40)	2 (20)	0.63	Fisher
Stage 4	4 (20)	1 (10)	3 (30)	0.58	Fisher
UPDRS	44.05 ± 15.97	41.8 ± 16.53	46.3 ± 15.93	0.26	U

Continuous variables are expressed as mean ± standard deviation; categorical variables are expressed as frequencies (%). Fisher (Fisher’s exact test); UPDRS (Unified Parkinson’s Disease Rating Scale); U (Mann–Whitney U test).

Exclusion criteria encompassed a history of significant neurological or psychiatric disorders unrelated to PD, the presence of severe sensory deficits, and cognitive impairment severe enough to interfere with task execution.

### 2.3 Procedures

As reported in the parent quasi-randomized clinical trial (16), the rehabilitation program lasted four weeks. Participants attended 12 rehabilitation sessions (three sessions per week over four weeks), each lasting approximately 45 min.

Clinical and instrumental assessments were performed at baseline (T0) and immediately after completion of the intervention (T1). T1 assessments were conducted within one week after the last rehabilitation session, in accordance with the study protocol described in the primary trial.

#### 2.3.1 Immersive virtual reality rehabilitation (CAREN system)

The rehabilitation protocol was implemented using CAREN, an advanced immersive VR system designed to provide multisensory feedback. Particularly, the CAREN system consists of a split-belt treadmill with force plate mounted on a motion platform, which allows controlled perturbations of balance, and a 180° projection screen that provides an engaging and ecologically valid training environment. During each sessionparticipants were engaged in a series of task-specific virtual scenarios aimed at improving gait adaptation, postural stability, and motor control. The environments were selected to progressively challenge motor function and included obstacle avoidance tasks requiring anticipatory and reactive motor planning, target stepping exercises to enhance gait initiation and precision, and balance perturbation challenges to promote dynamic postural adjustments. Throughout the training, participants received continuous visual and proprioceptive feedback, allowing for progressive adaptation and increased challenge levels based on their performance. A detailed description of the virtual scenarios used during the CAREN-based rehabilitation program, including task objectives, sensory features, and motor demands, is provided in the Supplementary Table S1.

#### 2.3.2 CAREN plus music

In the CAREN-M group, the immersive VR-based rehabilitation was combined with NMT sensory-motor techniques, specifically RAS and Therapeutic Instrumental Music Performance (TIMP), to enhance motor timing and coordination. It is important to clarify that both groups received the same rehabilitation dosage in terms of session number, session duration (approximately 45 min), and overall training intensity. The CAREN-M group did not perform additional exercises nor received longer training sessions. NMT techniques, specifically RAS and TIMP, were integrated within the same VR-based tasks performed by the CAREN group, replacing silence or non-rhythmic auditory conditions rather than increasing exercise time or volume. Therefore, the total amount of physical practice and task exposure was equivalent between groups, and any between-group differences cannot be attributed to differences in training dosage.

RAS is a structured auditory cueing technique that utilizes the natural rhythmic properties of the motor system to facilitate movement synchronization, improving gait parameters such as stride length, cadence, and step symmetry (27).

A certified NMT administrator delivered live rhythmic cues synchronized with each participant's natural walking pace. The auditory stimulus was dynamically adapted in real-time to optimize gait performance. The primary approach involved metronome-based entrainment, in which a steady rhythmic pulse, typically set between 1.0 and 1.2 Hz, was provided to regulate gait cadence. Additionally, live instrumentation was employed, incorporating acoustic markers such as guitar, tambourines, and maracas to reinforce step synchronization and provide real-time adaptability to individual gait patterns. In some sessions, TIMP techniques were introduced, enabling participants to actively engage in rhythmic cueing through the use of handheld percussive instruments, thereby enhancing sensorimotor integration.

To maximize the therapeutic effect, the time of rhythmic cues was initially matched to 90%–100% of each participant's preferred walking speed, then progressively increased by 5%–10% to encourage optimization of step length while maintaining stability. The music therapist continuously adjusted the rhythmic patterns based on real-time biomechanical feedback from the CAREN system, ensuring that participants maintained a stable and coordinated gait pattern throughout the intervention.

It should be emphasized that in the CAREN group, participants experienced auditory feedback inherent to the virtual environments (e.g., environmental sounds and non-rhythmic scenario audio provided by the CAREN software). These sounds were part of the immersive VR setup and corresponded to the selected virtual scenarios (e.g., ambient trail, interactive elements), without structured rhythmic or musical cues. In contrast, the CAREN-M group received NMT integration consisting of structured rhythmic auditory stimulation and live or recorded musical elements designed to provide external temporal cues during movement within the same scenarios. This combined approach of immersive VR and RAS was designed to amplify neuroplastic mechanisms by reinforcing sensorimotor integration, motor timing, and adaptive gait responses. Evidence from previous studies (21, 23, 28, 29) suggests that RAS-based interventions can improve stride symmetry and reduce gait variability in individuals with PD, supporting the hypothesis that integrating rhythmic auditory cues with immersive VR may provide an additive therapeutic benefit.

### 2.4 Clinical assessment

A skilled physiotherapist assessed motor performance in PD patients using specific clinical scales and tests selected to cover key domains relevant to gait rehabilitation in PD, including balance/fall risk, functional mobility, walking speed, fear of falling, and independence in activities of daily living. The Berg Balance Scale (BBS) evaluates static and dynamic balance across 14 tasks, with scores ranging from 0 to 56; scores below 40 indicate a moderate to high risk of falling (19). The Tinetti Scale (TS) consists of 16 items, 7 related to gait and 9 to balance, with a total score of 18 or less suggesting a high fall risk, and scores between 19 and 24 indicating a moderate risk (30). The Falls Efficacy Scale-International (FES-I) comprises 16 items measuring the perceived fear of falling, with total scores ranging from 16 (no fear) to 64 (strong fear) (31). BBS, TS and FES-I scores were normalized to a 0%–100% scale to allow direct comparison across clinical measures with different score ranges. Timed-up-and-go (TUG) consists of timing how long it takes a person to get up from a chair, walk 3 m, turn around and then sit down again, and it gives an estimation of risk of falls (32). The test was performed twice, once including a right turn and once including a left turn. These are reported as TUG R and TUG L, respectively, to quantify potential asymmetries in turning performance, which are clinically relevant in Parkinson's disease.; a PD patient who takes ≥ 11.5 s to complete the TUG is at risk for falling (33);10-meter walk test (10MWT) was used to assess walk speed in m/s over a short distance and the final score was obtained as the average of three trials. Although the 10MWT is commonly reported as walking speed (m/s), in this study we analyzed and presented the raw time required to walk 10 meters (in seconds), to maintain consistency with the other temporal clinical measures (e.g., TUG). This choice does not affect the interpretation of the test, as speed can be directly derived from the recorded time (34). Finally, the Barthel Index (BI) quantifies an individual's ability to perform basic activities of daily living independently, with scores from 0 (total dependent) to 100 (totally independent) (35).

### 2.5 Instrumental gait analysis

A skilled physiotherapist and a biomedical engineer evaluated patients' gait using the BTS Gaitlab system (BTS Bioengineering, Milan, Italy). This gait analysis system integrates a suite of tools that provide objective, quantitative assessments on subtle changes in posture, gait, load distribution, and muscle performance for clinical use. The BTS Gaitlab system included 8 infrared cameras (BTS SMART-DX) for three-dimensional motion capture, four BTS P-6000 force plates for kinetic analysis, and eight BTS FREEEMG 1000 wireless probes for surface electromyography.

Data acquisition and processing were performed using BTS SMART-Clinic software, which includes libraries of scientifically validated protocols (36, 37). In this study, we employed the “DAVIS Heel: multifactorial gait analysis” protocol to obtain quantitative data on kinematics, kinetics, and muscle activity, thereby assessing gait functionality. This protocol, which is based on the “Newington marker set” (36), required the measurement of each participant's anthropometric parameters, such as weight, height, tibia length, femoral condyle distance, knee and ankle diameters, iliac crest distance, and pelvis thickness.

The optoelectronic system captures kinematic data by tracking the positions of reflective markers placed on the patient's body, thereby calculating joint angles at the hip, knee, pelvis, trunk, and ankle in flexion-extension, abduction-adduction, and external-internal rotation. Gait events, such as initial ground contact and toe-off, are automatically identified via synchronized marker data, with subsequent manual verification by therapists through the software.

The recording protocol began with the patient standing in a neutral position for 5 s, followed by walking at a comfortable pace. Baseline evaluation was completed after an average of six acquisitions (mean ± SD = 2.5 ± 0.8 strides per acquisition). Simultaneously, EMG activity was recorded from eight wireless probes (four per limb) connected to the Smart Analyzer system (Version 1.10.469.0; BTS, Milan, Italy). In this study, EMG signals from the gastrocnemius lateralis, tibialis anterior, rectus femoris, and semitendinosus muscles were evaluated in accordance with European guidelines for surface EMG (SENIAM) (38). Before electrode placement, the skin was cleaned and dried, and bipolar surface electrodes were aligned with the direction of muscle fibers.

Normative data from a healthy adult cohort (40 participants: 28 males, 12 females, ages 18–40) (39, 40) were used for comparative analysis of kinematic and kinetic parameters, aiding in the identification of gait dysfunction.

Furthermore, data processed offline from the BTS Gaitlab system included additional parameters relevant to our analysis.

Kinematics: Spatio-temporal parameters, hip, knee, pelvis, trunk, and ankle joint angles in flexion-extension, abduction-adduction, and external-internal rotation measured during walking trials.

In particular, spatio-temporal parameters were recorded during the walking trial, with normative data obtained from the BTS Gaitlab. Temporal parameters included gait stride and stance duration (measured in seconds) as well as the percentages of the gait cycle allocated to stance, swing, single support, and double support phases. Spatial parameters comprised of stride length, step length, and step width (m), and spatio-temporal measures such as gait speed (m/s) were also analyzed. For each participant, data were averaged across both limbs and all gait strides. These parameters were then compared to the corresponding averages from healthy individuals to identify gait impairments. In addition, the R_CIRC_ symmetry index (Equation 1) (41) was employed to assess differences in joint rotation angles, expressed as a percentage of the gait cycle, thereby quantifying gait dysfunction by estimating deviations from normal gait patterns.

$$R_{\mathrm{CIRC}}=\frac{C_{\mathrm{xy}}}{\sqrt{\sum_{n=1}^{101}x{\left(n\right)}^{2}\sum_{n=1}^{101}y{\left(n\right)}^{2}}}$$ (1)

In this context, *x* represents the waveform for PD subjects, while y corresponds to the average waveform of the healthy population. C_xy_ denotes the circular cross-correlation function at lag 0, and the R_CIRC_ index ranges from −1 to 1, where a value of 1 indicates an identical amplitude profile shape.

Kinetics: Joint moments and powers at the hip, knee, and ankle joints.

Dynamic parameters, including joint moments and powers normalized to body weight (N·m/kg and W/kg, respectively) and ground reaction forces (GRF) expressed as a percentage of body weight, were averaged across all gait strides for each participant and across both limbs. These normalized dynamic data were then used to compute the R_CIRC_ shape symmetry index (Equation 1), which compares joint moments, powers, and GRF between pathological and healthy groups.

EMG: Muscle activation and co-activation patterns.

Muscle activation signals were recorded using surface electrodes to capture raw EMG signals (in mV). These signals were bandpass filtered between 20 and 450 Hz and normalized to the gait cycle duration (expressed as a percentage of the gait cycle). The signal amplitude, proportional to the muscle-generated force, was analyzed by calculating the root mean square (RMS) of the signal power within a moving window covering 10% of the gait cycle.

### 2.6 Statistical analysis

Sociodemographic and clinical variables were analyzed using descriptive statistics (mean ± SD for continuous, frequencies, and percentages for categorical data). Age, years of education, and UPDRS were treated as continuous; while gender and Hoehn & Yahr were considered categorical. Normality was assessed via the Shapiro–Wilk test (MATLAB function swtest). Given a normal distribution, age was compared between groups using a parametric t-test (ttest), while non-normal variables (education, UPDRS) were analyzed using the Mann–Whitney U test (ranksum). Categorical variables were evaluated with Fisher's exact test (fishertest).

Kinematic, kinetic, and EMG variables, as well as clinical outcome measures, were analyzed using non-parametric tests due to non-normal distributions. Between-group comparisons at T0 and T1 were performed using the Wilcoxon rank-sum test, while within-group changes (T0 vs. T1) were assessed with the Wilcoxon signed-rank test. Treatment-related changes were further evaluated by comparing T1–T0 deltas (*Δ*) between groups using the Mann–Whitney U test. For each variable, absolute values (median and interquartile range (IQR), mean and standard deviation (SD) were reported to allow interpretattion of change magnitude. All analyses were conducted in MATLAB R2022a (MathWorks, Natick, MA).

## 3 Results

**Figure 1:**
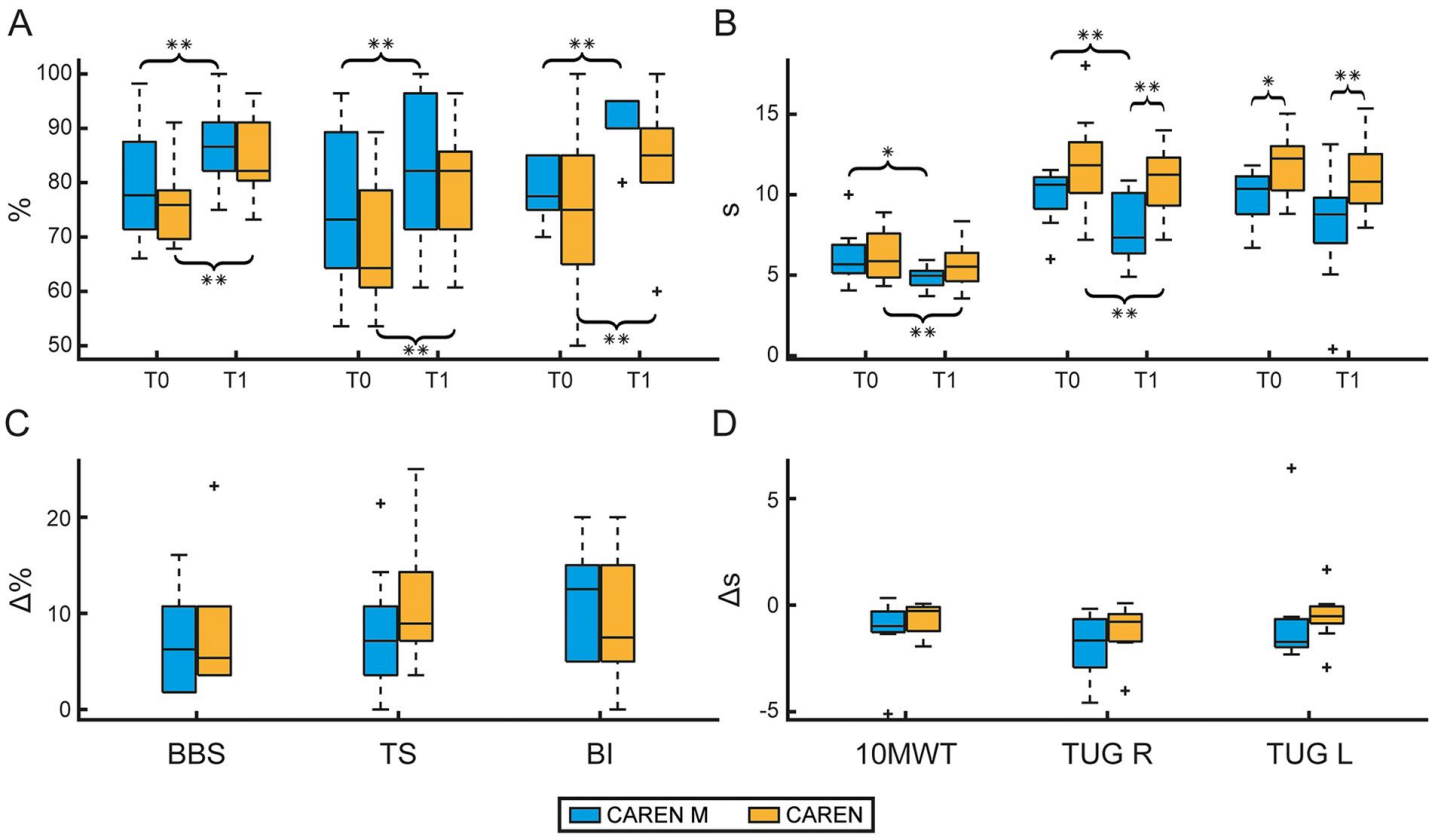
Clinical assessment. Boxplots display the outcomes of clinical scales for the CAREN M group (blue) and the CAREN group (orange) assessed at baseline (T0) and post treatment (T1). Panel **(A)** shows the normalized results (percentage, 0%–100%) for the Berg Balance Scale (BBS), Tinetti Scale (TS), and Barthel Index (BI). Panel **(B)** shows the outcomes for temporal clinical measures including the 10-meter walk test (10MWT), displayed as completion time (s), and the Time Up and Go (TUG) tests performed with a right turn (TUG R) and a left turn (TUG L) sides. Panels **(C and D)** show the delta values representing the absolute difference between T0 and T1 for each group and scale. Statistical difference significances are reported as *** < 0.001, ** < 0.01 and * < 0.05.

**Figure 2:**
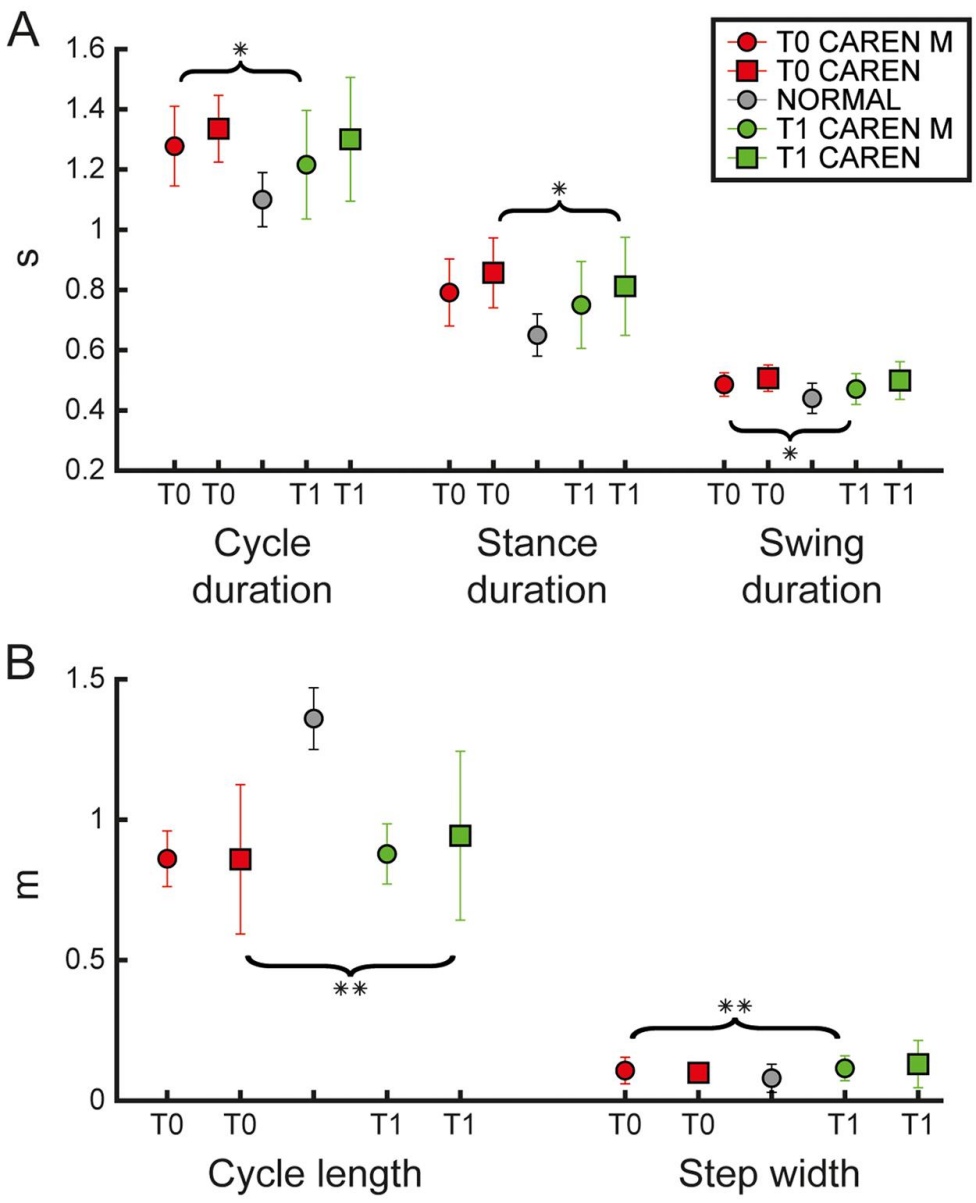
Kinematic parameters. The figure shows the kinematic parameters as mean ± standard deviation for each experimental group (CAREN M represented by circles, CAREN represented by squares, and Normal shown as grey circles) at baseline (T0, red) and post-treatment (T1, green). Panel **(A)** shows temporal kinematic parameters (cycle duration, stance duration, and swing duration). Panel **(B)** shows spatial kinematic parameters (stride length and step width). Statistical difference significance is reported as *** < 0.001, ** < 0.01 and * < 0.05.

**Figure 3:**
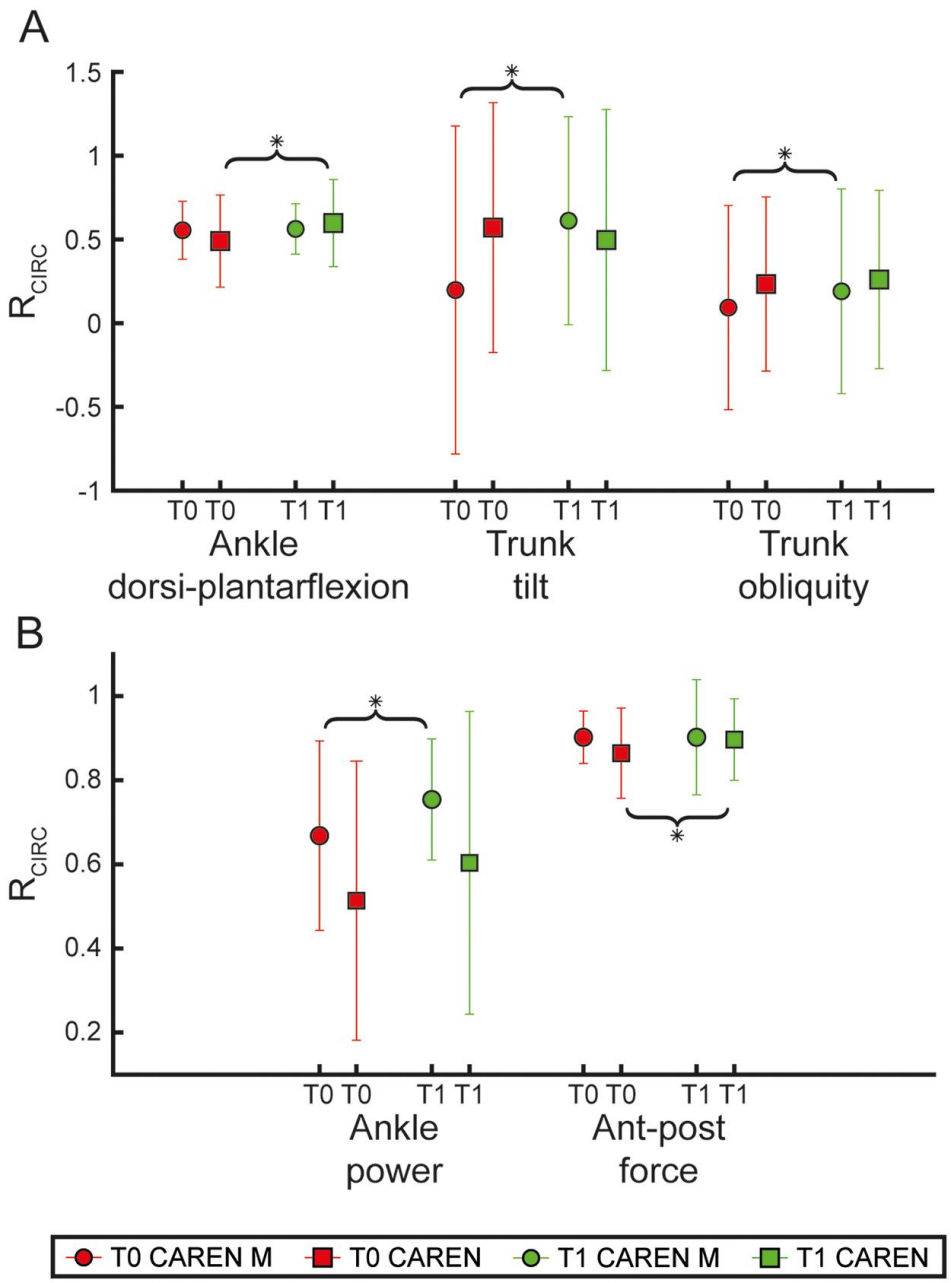
Kinematic and kinetic circular cross-correlation results. The figure shows the shape symmetry index R_CIRC_ for each experimental group (CAREN M represented by circles and CAREN represented by squares) at baseline (T0, red) and post treatment (T1, green). Panel **(A)** shows the R_CIRC_ index for the kinematic angles (ankle dorsi-plantarflexion, trunk tilt, trunk obliquity). Panel **(B)** shows R_CIRC_ index of the dynamic parameters, ankle power and anterior-posterior (Ant-post) force. Statistical difference significance is reported as *** < 0.001, ** < 0.01 and * < 0.05.

Baseline demographic and clinical characteristics of the CAREN and CAREN-M groups are summarized in Table 1. No statistically significant differences were observed between groups in terms of age, education, gender distribution, Hoehn & Yahr staging, or UPDRS-III scores. Significant improvements were observed in both clinical scales and instrumental outcomes for the CAREN group. Regarding clinical measures (see Table 2), there were significant improvements from baseline (T0) to post-treatment (T1) in BBS, TS, BI (Figures 1A, C), the 10MWT, and TUG R (Figures 1B, D), (*p* 0.002, *p* *=* 0.002, *p* = 0.008, *p* = 0.006, and *p* = 0.008, respectively). Regarding instrumental outcomes (see Supplementary Table S2), some temporal parameters showed significant improvements (Figure 2A). Specifically, stance duration and double support phase were significantly reduced from T0 to T1 (*p* = 0.021 and *p* = 0.044, respectively. Among spatial kinematic measures, cycle length (*p* = 0.004, Figure 2B) and gait speed (*p* = 0.001) both showed statistically significant improvements. Additionally, ankle dorsi-plantarflexion (Figure 3A) (assessed by the cross-correlation reconstruction index, R_CIRC_) improved significantly (*p* = 0.028). Dynamic analysis (Figure 3B) revealed a significant increase in anterior-posterior force R_CIRC_ (*p* = 0.043), and EMG recordings showed significant increases in the RMS values of the tibialis anterior, gastrocnemius muscles and rectus femoris (*p* = 0.006, *p* = 0.037 and *p* = 0.010, respectively).

**Table 2:** Clinical outcomes.

Variables	CAREN M T0	CAREN M T1	CAREN M *Δ*	CAREN T0	CAREN T1	CAREN *Δ*	*p* CAREN M (T0 VS T1)	*p* CAREN (T0 VS T1)	*p* T0 CAREN M VS CAREN	*p* T1 CAREN M VS CAREN	*P Δ* CAREN M VS CAREN
BBS (%)	77.7 [71.4–87.5]	86.6 [82.1–91.1]	6.2 [1.8–10.7]	75.9 [69.6–78.6]	82.1 [80.4–91.1]	5.4 [3.6–10.7]	**0**.**002**	**0**.**002**	0.477	0.559	0.731
TS (%)	73.2 [64.3–89.3]	82.1 [71.4–96.4]	7.1 [3.6–10.7]	64.3 [60.7–78.6]	82.1 [71.4–85.7]	8.9 [7.1–14.3]	**0**.**004**	**0**.**002**	0.551	0.914	0.208
FES-I (%)	15.6 [10.4–29.2]	11.5 [8.3–18.8]	−2.1 [−6.3–0.0]	17.7 [10.4–39.6]	13.5 [6.2–33.3]	−2.1 [−6.3–0.0]	0.121	0.477	0.607	0.496	0.733
BI (%)	77.5 [75.0–85.0]	90.0 [90.0–95.0]	12.5 [5.0–15.0]	75.0 [65.0–85.0]	85.0 [80.0–90.0]	7.5 [5.0–15.0]	**0**.**002**	**0**.**008**	0.656	0.090	0.332
10MWT (s)	5.7 [5.1–6.9]	5.0 [4.4–5.3]	−1.0 [−1.3–−0.3]	5.9 [4.9–7.6]	5.5 [4.6–6.4]	−0.3 [−1.2–−0.1]	**0**.**010**	**0**.**006**	0.846	0.131	0.273
TUG R (s)	10.6 [9.1–11.1]	7.3 [6.4–10.1]	−1.7 [−2.9–−0.7]	11.8 [10.1–13.3]	11.2 [9.3–12.3]	−0.8 [−1.7–−0.4]	**0**.**002**	**0**.**008**	0.105	**0**.**002**	0.186
TUG L (s)	10.4 [8.8–11.1]	9.2 [7.6–9.8]	−1.7 [−2.0–−0.7]	12.2 [10.3–13.0]	10.8 [9.5–12.5]	−0.5 [−0.9–−0.1]	0.084	0.084	**0**.**020**	**0**.**002**	0.070

Clinical scale scores for the CAREN M and CAREN groups at baseline (T0), post-treatment (T1), and treatment-related changes (*Δ* = T1–T0) are reported. Data are expressed as median and interquartile range [IQR]. Within-group comparisons (T0 vs. T1) were performed using the Wilcoxon signed-rank test, between-group comparisons at T0 and T1 using the Wilcoxon rank-sum test, and between-group comparisons of *Δ* using the Mann–Whitney U test. Statistically significant *p*-values are shown in bold. Scores for BBS, TS, FES-I, and BI are reported as percentages (0%–100%). TUG and 10MWT values are reported in seconds. TUG R and TUG L indicate the Timed Up and Go test performed with a right and left turning direction, respectively.

BBS, Berg Balance Scale; TS, Tinetti scale; FES-I, Falls Efficacy Scale – International; BI, Barthel Index; 10MWT, 10 meters walking test; TUG R/L, Timed-UP-Go right/left.

In the CAREN-M group, significant improvements were also observed in both clinical scales (Table 2) and instrumental outcomes (Supplementary Table S2). Clinical scales (BBS, TS, BI, 10MWT, and TUG R) improved significantly from T0 to T1 (*p* = 0.002, *p* = 0.004, *p* = 0.002, *p* = 0.010, and *p* = 0.002, respectively, see Figure 1). Instrumental outcomes demonstrated significant enhancements in some temporal kinematic parameters both from pre- to post-treatment and relative to normative values. Cycle duration and swing duration were significantly reduced from T0 to T1 (*p* = 0.035 and *p* = 0.019, respectively, Figure 2A). Among spatial kinematic measures (Figure 2B), step width significantly increased from T0 to T1 (*p* = 0.005). Moreover, trunk tilt and obliquity R_CIRC_ (Figure 3A) improved significantly between T0 and T1 (*p* = 0.030 and *p* = 0.040, respectively), as did the dynamic parameter ankle power R_CIRC_ (*p* = 0.048).

At baseline (T0), groups did not differ in TUG R (*p* = 0.105), whereas a difference was observed for TUG L (*p* = 0.020). From T0 to T1, both groups improved TUG performance; the temporal differences *Δ* were −1.7 [−2.9 to −0.7] s (R) and −1.7 [−2.0 to −0.7] s (L) in the CAREN M group and −0.8 [−1.7 to −0.4] s (R) and −0.5 [−0.9 to −0.1] s (L) in the CAREN group. At T1, between-group differences were statistically significant for both turning directions (TUG R: *p* = 0.002; TUG L: *p* = 0.002).

Direct comparison of treatment-induced changes *Δ* revealed significant between-group differences for specific parameters. The CAREN group showed a greater improvement in cycle length compared to the CAREN M group (*p* = 0.048), whereas the CAREN M group exhibited a larger improvement in trunk tilt R_CIRC_ (*p* = 0.036). Additionally, rectus femoris RMS increased significantly more in the CAREN group than in the CAREN M group (*p* = 0.031).

## 4 Discussion

In this study, we explored the effectsof two different protocols using immersive VR-based rehabilitation interventions, the standalone VR training with CAREN system and the CAREN system in addition to NMT, in improving gait and balance functions in PD patients. Both interventions led to improvements from T0 to T1 across balance, functional mobility, and activities of daily living (BBS, TS, TUG, 10MWT, and BI), together with favorable changes in instrumental gait parameters. In real-world scenarios, these changes may translate into safer transfers and turning, faster and more stable walking, and greater independence during everyday activities.

### 4.1 Clinical findings

In terms of clinical motor outcomes, the CAREN group demonstrated significant improvements in both static and dynamic balance, as measured by the BBS, indicating enhanced postural stability. These findings are consistent with previous studies showing that VR can improve balance in patients with neurological disorders by immersing them in environments that stimulate proprioceptive feedback and motor control (11, 13, 25, 42–44). Additional clinical improvements were observed, particularly in gait mobility (TUG, TS) and functional independence (BI), suggesting increased safety during ambulation. VR supports the repetition of specific, individualized tasks, which is an essential factor in restoring motor function and reducing disability (45). Supporting these results, Pullia et al. (25) reported that semi-immersive VR-based treadmill training (C-Mill, Motek, Netherlands) in patients with PD led to superior outcomes in gait and balance performance (TS, BBS, and 6MWT), reduced fear of falling, and improved overall independence.

Although both groups showed comparable improvements in clinical motor scales, suggesting that immersive VR-based rehabilitation *per se* is effective in enhancing balance, gait, and functional mobility in people with PD, the integration of NMT in CAREN-M group appears to modulate the qualitative profile of motor improvement rather than its overall magnitude. In particular, while the CAREN group primarily improved spatial and distal motor components of gait (e.g., gait speed, stride-related parameters, ankle kinematics), the CAREN-M group exhibited a more pronounced improvement in temporal gait organization and trunk control, which are clinically relevant for turning and dynamic postural stability.

Consistently, the CAREN-M group also improved across clinical outcomes (Table 2), supporting feasibility of integrating NMT within CAREN. These improvements highlight the potential role of NMT to significantly potentiate motor control and functional abilities in patients with neurological disorders, as shown by similar studies on the therapeutic benefits of synchronized movement and music in rehabilitation (24, 28, 46–49).

This interpretation is supported by turning-related outcomes. Despite similar within-group improvements in TUG performance, between-group comparisons at post-treatment favored the CAREN-M group, suggesting that RAS may facilitate movement sequencing and timing during complex motor tasks such as turning. From a clinical perspective, this finding is particularly relevant in Parkinson's disease, where impaired temporal organization of gait and turning is closely linked to fall risk and functional disability. In our study, both groups (CAREN and CAREN-M) showed a statistically significant improvement from T0 to T1 in TUG performed with a right turn (TUG R), whereas the change did not reach statistical significance for the left-turn condition (TUG L). This direction-specific pattern may reflect the asymmetric nature of motor impairment in PD (50, 51). In addition, turning performance may differ between directions and show substantial between-subject variability (50, 52), which could limit the ability to detect within-group change in small samples. Importantly, groups already differed at baseline for TUG L, suggesting an initial imbalance in left-turn performance that may have reduced the interpretability of post-intervention comparisons for this condition.

Finally, the significant between-group differences observed at T1 for both turning directions indicate that CAREN-M achieved overall better TUG performance at post-treatment; however, given the baseline difference in TUG L, these findings should be interpreted cautiously and ideally confirmed in larger samples, possibly stratifying participants by the clinically more affected side and quantifying direction-specific freezing/turning difficulty. Notably, this direction-specific improvement in turning-related functional mobility is consistent with evidence that rhythmic auditory cueing can acutely enhance functional turning speed in people with PD, likely by supporting attentional control during turning (53).

Overall, auditory cueing within the CAREN-M protocol may not primarily increase global clinical scores, but rather refine gait timing, coordination, and trunk/postural control, which are particularly relevant for complex tasks such as turning and dynamic balance (20, 54).

Different from other immersive VR systems, CAREN integrates a 6-degree-of-freedom instrumented platform with a belt treadmill, delivering rich sensorimotor stimulation to the brain that further enhances the effects of the targeted stimuli. Indeed, treadmill walking alone has been shown to produce lasting gains in a number of gait parameters, likely through its impact on some of the neuroplastic processes in the complicated cortical–basal ganglia–cerebellar networks. Adding auditory cues, though, may produce additional gait benefits by introducing an external temporal scaffold compensates for impaired internal timing mechanisms, a core feature of Parkinsonian gait dysfunction (28, 54, 55).

This effect is thought to be mediated by the activation of frontoparietal networks and their crosstalk with cortico–basal ganglia–cerebellar circuits, thereby facilitating the generation of automatic and rhythmic movement when intrinsic timing mechanisms are compromised (56, 57). In this context, NMT has been shown to refine the temporal organization and consistency of gait rather than simply increasing overall motor output, an effect that may not always be fully captured by global clinical scales (24, 27, 48). This hypothesis has been demonstrated by the neurophysiological study by Calabrò et al. (21), who found that NMT-based auditory cueing can target motor cortical beta-band frequency range synchrony during steady-state treadmill walking in patients with PD., suggesting a direct modulation of motor timing and coordination. Converging findings have been reported in electrophysiological studies involving individuals with Parkinson's disease undergoing deep brain stimulation of the subthalamic nucleus, in which rhythmic auditory cueing increased beta-band modulation (28–30 Hz), reduced step-timing variability, and strengthened the coupling between beta activity and step timing, all markers of a more regular and stable gait pattern (58).

Moreover, recent experimental and theoretical work suggests that beta-band modulation represents one of the key neural mechanisms through which rhythmic auditory stimulation facilitates gait cadence, temporal stability, and coordination in PD (48, 59).

### 4.2 Instrumental findings

Together with clinical motor improvements, we registered positive changes in various gait parameters, demonstrating the potential effects of both interventions in enhancing motor functions. In particular, we registered a notable reduction in stance duration and double support phase (Supplementary Table S2) in the CAREN group alone, indicating an increase in gait efficiency as well as improved balance.

Significant improvements in cycle length and gait speed were observed. These two parameters are closely related, particularly in terms of the mobility function. In this context, Bytyçi et al. (60), in their meta-analysis, reported that gait speed is associated with better survival rates among older adults as well as reflecting overall health and functional status. For instance, shorter stride length combined with higher stride length variability are considered key factors contributing to poor balance, increased physical disability, and other adverse clinical outcomes.

Moreover, we found that the VR training with the CAREN system alone (CAREN group) significantly improved ankle dorsi-plantarflexion, which suggests increased mobility. This finding aligns with EMG results, which revealed an increased muscle activation intensity (RMS) in the Tibialis Anterior and Gastrocnemius muscles. Improved ankle control may contribute to a more efficient and coordinated gait pattern (61, 62), ultimately reducing the risk of falls and enhancing overall functional independence in individuals with PD. In addition, we found an improvement in the Rectus Femoris after the training with the CAREN alone (63, 64), indicating better muscle recruitment and motor adaptation after the training.

Regarding the CAREN M group, patients showed improvements in some kinematic temporal parameters, including cycle duration and swing duration. Consistent with our findings, previous research has shown that RAS can effectively modify gait biomechanics in PD patients by enhancing temporal and spatial parameters of gait (55, 65, 66). Lin et al. (67) similarly reported RAS-related changes in spatiotemporal gait parameters in individuals with PD, including gait speed, stride length, and swing time. Overall, these findings support the notion that auditory cueing can modulate locomotor patterns by leveraging relatively preserved auditory–motor coupling in PD (20, 68). From a biomechanical standpoint, gait-cycle duration reflects the timing of the entire gait sequence and is strongly influenced by the duration of its subcomponents, including swing. Therefore, reductions in swing duration are expected to contribute to a shorter overall cycle duration, particularly when accompanied by an increase in walking speed. In addition, consistent with Pau et al. (69), we observed a significant increase in step width. The authors interpreted this change as a potential “side effect” of the intervention, as higher step-width values are often associated with reduced stability and fear of falling (70, 71). It has been hypothesized that this effect may be related to the increase in walking speed (i.e., shorter stride and swing durations), suggesting that patients adapted their gait strategy to higher speed by widening their step width (69). This interpretation is also consistent with evidence that individuals with PD and FOG may rely on compensatory postural strategies to help preserve dynamic stability during gait (72).

Moreover, improvements in trunk tilt and obliquity, alongside enhancements in temporal and spatial gait kinematics, further indicate enhanced postural control and lower limb mechanics (54). It is worth noting that individuals with PD often manifest a reduction in motor automaticity, leading to fragmented movements, such as turning with a “block-like” motion (turning in block) and challenges with initiating steps (3, 73). Our findings collectively suggest that the role of RAS in optimizing movement timing and coordination, addressing some of the most challenging aspects of PD (55, 74). Rhythmic cues facilitate motor entrainment, enabling patients to synchronize their movements with external auditory stimuli, thereby enhancing movement fluidity and reducing temporal deviations (47, 55).

The observed differences in outcomes between the CAREN and CAREN-M groups can be elucidated through the neuroplastic mechanisms invoked by the multisensory integration of auditory and visual stimuli. Rhythmic feedback is known to intensify neuronal activity in brain areas involved in motion planning and execution, which could directly influence motor circuits impaired by PD (59, 75, 76). Another possible explanation for the differences in our findings, regarding the two groups, may rely on the different components of gait control. The CAREN alone training relies primarily on immersive visuomotor and proprioceptive feedback within goal-directed tasks (e.g., obstacle negotiation, target stepping, balance perturbation challenges) (77, 78), which may lead adaptations in gait kinematics and distal joint mechanics (79, 80). This interpretation is consistent with the larger improvements observed in spatial parameters (e.g., gait speed, cycle length) and in ankle-related kinematics/kinetics, together with increased lower-limb muscle activation (EMG RMS), which may reflect improved neuromuscular coordination (81, 82). By contrast, adding RAS in the CAREN-M protocol provides an external temporal stimulus that may facilitate motor entrainment and improve timing and coordination of gait cycles (83). In this context, RAS may primarily influence temporal organization (e.g., cycle and swing duration) (69, 84) and contribute to more stable trunk control (tilt/obliquity), potentially by promoting a more consistent rhythm and reducing temporal irregularities during walking (85).

### 4.3 Limitation

This study is a secondary analysis based on a subset of participants with complete motor outcome data, which may introduce selection bias and limit statistical power, thereby reducing generalizability. Furthermore, the small sample size and the quasi-randomized study design may have limited statistical power and increased the risk of selection bias and residual confounding. In addition, the third study arm involving conventional physiotherapy, originally planned, was excluded because substantial baseline heterogeneity in clinical measures would have undermined the validity of comparisons. Although baseline differences in age and Hoehn & Yahr stage between groups were not statistically significant, they may still be clinically meaningful in a small sample and could have influenced responsiveness to training. Age and disease severity (including Hoehn & Yahr stage) have been reported as predictors of gait improvements following rehabilitation in PD, with younger and less severely affected individuals being more likely to show gains (86). Moreover, disease severity has also been shown to exert a substantial effect on spatial gait features (e.g., step length) even after controlling age (87). Finally, motivational factors were not assessed in our study; however, observational evidence suggests that exercise motivation in PD may be lower with increasing age and higher perceived disease severity, which could act as an unmeasured confounder (88). Therefore, our between-group comparisons should be interpreted cautiously, and future studies should consider baseline-adjusted analyses and/or stratification by age and disease severity.

## 5 Conclusion

In this secondary analysis, both VR-based rehabilitation protocols were associated with improvements in gait and postural performance in patients with PD. Notably, the CAREN system positively influenced clinical motor scores and instrumental outcomes. Specifically, the CAREN group exhibited significant improvements in spatial kinematic and neurophysiological parameters, while the CAREN-M group, which integrated NMT, showed greater enhancement in temporal gait parameters and trunk stability. This suggests a more holistic and synergistic rehabilitation approach. Further research is warranted to evaluate the long-term effects, individual adaptability, and personalized implementation of such interventions across different stages of disease progression.

## Data Availability

The raw data supporting the conclusions of this article will be made available by the authors, without undue reservation.
