# Supplementary material for: Can neurologic music therapy make the difference when using immersive virtual reality in Parkinson disease motor training? Promising findings from a secondary analysis
Source: Front Rehabil Sci. 2026 Feb 26;7:1707528. doi: 10.3389/fresc.2026.1707528 (PMC12979535; doi:10.3389/fresc.2026.1707528)
Supplement: Supplementary file 1 [file Table1.docx]

**Supplementary Table S1**: description of CAREN scenarios with target skills, description of the scenarios and their training goals, lastly NMT procedure used for CAREN M group.

| VR Scenario | Skills | Description | Training goal | NMT Procedure |
| --- | --- | --- | --- | --- |
| Italian Alps | Gait  Gait Stability  Balance  Motor Abilities  Executive Functions  Attention  Visuo-spatial abilities | Challenging patient with guiding a cart through obstacle courses, traversing varying terrain gradients, and maximizing pizza assembly by gathering ingredients distributed along the pathway. Ingredients are located on the road, allowing the patient to collect them by steering the cart over them while navigating around obstacles. | Provide training sessions focusing on uphill and downhill walking, combined with an adaptability slalom task, aimed at enhancing gait stability and boosting physical capacity. | The Therapist provided live music on which they walked while retrieving the pizza ingredients A combination of techniques such as RAS and TIMP were used, along with the addition of musical instruments (maracas and tambourines) that were used concurrently with the arrival of stimuli (pizza ingredients) in addition to musical bases |
| Step on it | Gait  Gait stability,  Gait Adaptability  Paced Rhythm  Attention  Executive Functions  Visuo-spatial abilities | The participant walks along an unending pathway observed from a top view. Step length and width are assessed during gait, with cues generated in accordance with the participant's walking pattern. | The goal of this training is to challenge patients with adapting their step width and length, consequently altering step frequency in response to environmental cues. The uncluttered and straightforward virtual environment renders this application suitable for individuals with both physical and cognitive impairments. | Live accompaniment was played with the acoustic guitar while the patient walks. RAS was used to enhance rhythmic engagement and improve gait synchronization with the music |
| Road Encounters | Balance,  Gait stability,  Physical and cognitive Dual Task  Attention  Executive Functions  Visuo-spatial abilities | The participant walks over a rough and twisting forest trail, hitting insects and birds in flight with her/his hands. Walking on the treadmill occurs either at a set speed or under the control of a self-paced algorithm. | Task the patient with engaging in a physical dual-task, involving upper extremity movements simultaneous to walking along a winding and hilly forest trail. | Live music was played and the patient, while walking to the beat of the music, had to interact with the birds and butterflies in the virtual scenario, and when it was time to hit them, they also hit the tambourine or shook the maracas provided. A combination of techniques such as RAS and TIMP were used |
| Rope | Gait,  Gait Stability,  Gait Adaptability  Physical and cognitive Dual Task  Attention  Executive Functions  Visuo-spatial abilities | The participant walks on a suspended rope bridge between towers, encountering gusts of wind that induce platform sway perturbations. While navigating the bridge, seagulls may fly past and must be evaded | This application simulates the complexity of outdoor walking within the safe and controlled environment of the treadmill system. Platform and treadmill mimic the slope and sway of the bridge, utilizing pitch and sway adjustments Anticipation training is achieved by avoiding the seagulls flying. | Live music with rhythm suitable for the pace the patient had to keep was sent. RAS was used to choose the right tempo that matches the speed of the movement you want to encourage, while TIMP technique was employed to better coordinate the patient's movements to avoid the obstacles (seagulls) along the path, using a tambourine or maracas while moving the treadmill to the left or right. |
| Stroop and Arithmetic | Gait,  Gait Adaptability  Cognitive Dual Task  Attention  Executive Functions | The participant walks on the treadmill, either maintaining a constant speed or under the control of a self-paced algorithm. Depending on the chosen task a coloured word or simple calculations are displayed followed by the correct answer. | Engage the patient in a cognitive dual task by incorporating the STROOP test or arithmetic exercises during gait. | Only the stroop part was selected; the patient had to walk in time with the live music played by the therapist and respond to the stimuli presented on the screen. RAS was employed to boost rhythmic involvement and enhance coordination of gait with the music |
