# Supplementary material for: Can neurologic music therapy make the difference when using immersive virtual reality in Parkinson disease motor training? Promising findings from a secondary analysis
Source: Front Rehabil Sci. 2026 Feb 26;7:1707528. doi: 10.3389/fresc.2026.1707528 (PMC12979535; doi:10.3389/fresc.2026.1707528)
Supplement: Supplementary file 2 [file Table2.docx]

**Supplementary Table S2. Instrumental outcomes.** Spatiotemporal, kinematic, kinetic, and EMG parameters for CAREN M and CAREN groups at T0, T1, and Δ (T1–T0). Data are expressed as mean ± SD. Within-group comparisons were performed using the Wilcoxon signed-rank test; between-group comparisons at T0 and T1 using the Wilcoxon rank-sum test; Δ comparisons using the Mann–Whitney U test. Statistically significant p-values are shown in bold. R_CIRC_: cross-correlation reconstruction index; RMS: root mean square.

| Variables | CAREN M  T0 | CAREN M  T1 | CAREN M  Δ | CAREN  T0 | CAREN  T1 | CAREN  Δ | p CAREN M  (T0 VS T1) | p CAREN  (T0–T1) | p T0  CAREN M vs CAREN | p T1  CAREN M vs CAREN | p Δ  CAREN M vs CAREN |
| --- | --- | --- | --- | --- | --- | --- | --- | --- | --- | --- | --- |
| Cycle duration (s) | 1.3 ± 0.1 | 1.2 ± 0.2 | -0.1 ± 0.1 | 1.3 ± 0.1 | 1.3 ± 0.2 | -0.0 ± 0.2 | **0.035** | 0.116 | 0.489 | 0.627 | 0.882 |
| Stance duration (s) | 0.8 ± 0.1 | 0.8 ± 0.1 | -0.0 ± 0.1 | 0.9 ± 0.1 | 0.8 ± 0.2 | -0.0 ± 0.1 | 0.070 | **0.021** | 0.227 | 0.779 | 0.424 |
| Swing duration (s) | 0.5 ± 0.0 | 0.5 ± 0.1 | -0.0 ± 0.0 | 0.5 ± 0.0 | 0.5 ± 0.1 | -0.0 ± 0.1 | **0.019** | 0.151 | 0.155 | 0.432 | 0.684 |
| Stance phase (%) | 61.8 ± 2.8 | 61.3 ± 3.1 | -0.5 ± 2.2 | 64.2 ± 4.8 | 62.2 ± 3.4 | -2.0 ± 4.5 | 0.113 | 0.057 | 0.179 | 0.526 | 0.499 |
| Swing phase (%) | 38.3 ± 3.0 | 39.1 ± 2.9 | 0.8 ± 1.8 | 38.4 ± 3.3 | 38.5 ± 2.9 | 0.1 ± 2.5 | 0.062 | 0.455 | 0.709 | 0.627 | 0.482 |
| Single support phase (%) | 38.3 ± 2.8 | 39.1 ± 2.9 | 0.8 ± 1.8 | 38.5 ± 3.9 | 38.6 ± 3.0 | 0.1 ± 3.1 | 0.093 | 0.411 | 0.852 | 0.627 | 0.882 |
| Double support phase (%) | 11.9 ± 2.4 | 11.3 ± 2.9 | -0.5 ± 2.0 | 13.5 ± 3.5 | 12.0 ± 3.1 | -1.5 ± 2.8 | 0.313 | **0.044** | 0.086 | 0.654 | 0.310 |
| Mean speed (m/s) | 0.68 ± 0.13 | 0.75 ± 0.15 | 0.07 ± 0.15 | 0.66 ± 0.23 | 0.76 ± 0.26 | 0.10 ± 0.11 | 0.062 | **0.001** | 0.572 | 0.982 | 0.303 |
| Cadence (steps/min) | 95.3 ± 10.3 | 101.0 ± 15.5 | 5.7 ± 8.5 | 91.4 ± 6.9 | 94.2 ± 11.7 | 2.8 ± 8.9 | 0.067 | 0.112 | 0.501 | 0.601 | 0.598 |
| Cycle length (m) | 0.86 ± 0.10 | 0.88 ± 0.11 | 0.02 ± 0.11 | 0.86 ± 0.27 | 0.94 ± 0.30 | 0.08 ± 0.11 | 0.556 | **0.004** | 0.354 | 0.575 | **0.048** |
| Step length (m) | 0.43 ± 0.06 | 0.43 ± 0.06 | 0.00 ± 0.05 | 0.40 ± 0.18 | 0.41 ± 0.14 | 0.01 ± 0.21 | 0.812 | 0.091 | 0.332 | 0.384 | 0.093 |
| Step width (m) | 0.11 ± 0.05 | 0.12 ± 0.04 | 0.01 ± 0.01 | 0.10 ± 0.03 | 0.13 ± 0.08 | 0.03 ± 0.08 | **0.005** | 0.466 | 0.645 | 0.941 | 0.214 |
| Pelvic obliquity (R_CIRC_) | 0.140 ± 0.414 | 0.143 ± 0.471 | 0.003 ± 0.513 | -0.070 ± 0.511 | 0.082 ± 0.518 | 0.152 ± 0.411 | 0.970 | 0.145 | 0.093 | 0.737 | 0.298 |
| Pelvic tilt (R_CIRC_) | 0.996 ± 0.004 | 0.995 ± 0.008 | -0.001 ± 0.009 | 0.818 ± 0.551 | 0.766 ± 0.607 | -0.052 ± 0.868 | 0.191 | 0.737 | 0.575 | 0.145 | 0.394 |
| Pelvic rotation (R_CIRC_) | 0.336 ± 0.274 | 0.262 ± 0.291 | -0.074 ± 0.179 | 0.251 ± 0.421 | 0.248 ± 0.424 | -0.003 ± 0.268 | 0.093 | 0.970 | 0.296 | 0.823 | 0.262 |
| Hip abduction-adduction (R_CIRC_) | 0.598 ± 0.202 | 0.668 ± 0.188 | 0.069 ± 0.275 | 0.467 ± 0.230 | 0.545 ± 0.254 | 0.078 ± 0.169 | 0.411 | 0.204 | **0.048** | 0.156 | 0.756 |
| Hip flexion–extension (R_CIRC_) | 0.968 ± 0.030 | 0.963 ± 0.022 | -0.005 ± 0.022 | 0.935 ± 0.054 | 0.955 ± 0.034 | 0.020 ± 0.053 | 0.370 | 0.204 | **0.007** | 0.709 | 0.208 |
| Hip rotation (R_CIRC_) | 0.327 ± 0.322 | 0.245 ± 0.369 | -0.082 ± 0.460 | 0.246 ± 0.388 | 0.234 ± 0.364 | -0.012 ± 0.331 | 0.575 | 0.940 | 0.575 | 0.823 | 0.839 |
| Knee flexion–extension (R_CIRC_) | 0.960 ± 0.038 | 0.961 ± 0.022 | 0.001 ± 0.033 | 0.931 ± 0.064 | 0.932 ± 0.062 | 0.001 ± 0.065 | 0.575 | 0.970 | **0.030** | 0.204 | 0.776 |
| Ankle dorsi-plantarflexion (R_CIRC_) | 0.555 ± 0.173 | 0.563 ± 0.150 | 0.008 ± 0.141 | 0.490 ± 0.275 | 0.598 ± 0.260 | 0.108 ± 0.167 | 0.852 | **0.028** | 0.332 | 0.526 | 0.108 |
| Trunk tilt (R_CIRC_) | 0.198 ± 0.980 | 0.612 ± 0.621 | 0.414 ± 0.944 | 0.571 ± 0.747 | 0.497 ± 0.780 | -0.074 ± 0.761 | **0.030** | 0.681 | 0.550 | 0.370 | **0.036** |
| Trunk obliquity (R_CIRC_) | 0.093 ± 0.610 | 0.190 ± 0.611 | 0.097 ± 0.249 | 0.234 ± 0.520 | 0.261 ± 0.532 | 0.027 ± 0.361 | **0.040** | 0.575 | 0.332 | 0.478 | 0.525 |
| Trunk rotation (R_CIRC_) | 0.422 ± 0.472 | 0.381 ± 0.481 | -0.041 ± 0.198 | 0.342 ± 0.487 | 0.379 ± 0.499 | 0.036 ± 0.362 | 0.654 | 0.433 | 0.351 | 0.681 | 0.379 |
| Knee varus–valgus (R_CIRC_) | 0.545 ± 0.570 | 0.503 ± 0.644 | -0.042 ± 0.646 | 0.574 ± 0.534 | 0.744 ± 0.398 | 0.170 ± 0.703 | 0.654 | 0.062 | 0.737 | 0.126 | 0.126 |
| Knee rotation (R_CIRC_) | 0.181 ± 0.633 | 0.283 ± 0.650 | 0.102 ± 0.946 | 0.195 ± 0.627 | 0.483 ± 0.529 | 0.287 ± 0.797 | 0.411 | 0.126 | 0.852 | 0.204 | 0.715 |
| Hip moment (R_CIRC_) | 0.665 ± 0.265 | 0.699 ± 0.171 | 0.034 ± 0.320 | 0.625 ± 0.349 | 0.713 ± 0.286 | 0.088 ± 0.323 | 0.913 | 0.396 | 0.586 | 0.528 | 0.609 |
| Knee moment (R_CIRC_) | 0.355 ± 0.251 | 0.381 ± 0.274 | 0.026 ± 0.271 | 0.382 ± 0.289 | 0.415 ± 0.276 | 0.032 ± 0.334 | 0.586 | 0.913 | 0.616 | 0.711 | 0.737 |
| Ankle moment (R_CIRC_) | 0.951 ± 0.042 | 0.963 ± 0.034 | 0.012 ± 0.043 | 0.951 ± 0.054 | 0.946 ± 0.071 | -0.005 ± 0.079 | 0.122 | 0.879 | 0.231 | 0.248 | 0.286 |
| Hip power (R_CIRC_) | 0.481 ± 0.322 | 0.531 ± 0.213 | 0.050 ± 0.367 | 0.497 ± 0.292 | 0.536 ± 0.316 | 0.038 ± 0.251 | 0.679 | 0.528 | 0.372 | 0.500 | 0.965 |
| Knee power (R_CIRC_) | 0.324 ± 0.343 | 0.281 ± 0.480 | -0.043 ± 0.498 | 0.420 ± 0.426 | 0.352 ± 0.463 | -0.069 ± 0.529 | 0.983 | 0.396 | 0.557 | 0.948 | 0.530 |
| Ankle power (R_CIRC_) | 0.668 ± 0.225 | 0.754 ± 0.144 | 0.086 ± 0.153 | 0.513 ± 0.332 | 0.604 ± 0.360 | 0.090 ± 0.205 | **0.048** | 0.078 | 0.267 | 0.372 | 0.942 |
| Anterior–posterior force (R_CIRC_) | 0.902 ± 0.062 | 0.901 ± 0.137 | -0.001 ± 0.104 | 0.864 ± 0.107 | 0.897 ± 0.097 | 0.032 ± 0.109 | 0.199 | **0.043** | 0.913 | 0.557 | 0.203 |
| Medio–lateral force (R_CIRC_) | 0.929 ± 0.024 | 0.938 ± 0.027 | 0.008 ± 0.018 | 0.921 ± 0.047 | 0.892 ± 0.162 | -0.029 ± 0.151 | 0.133 | 0.845 | 0.777 | 0.913 | 0.511 |
| Vertical force (R_CIRC_) | 0.975 ± 0.017 | 0.979 ± 0.014 | 0.004 ± 0.011 | 0.974 ± 0.014 | 0.973 ± 0.027 | -0.001 ± 0.030 | 0.248 | 0.306 | 0.557 | 0.616 | 0.609 |
| Tibialis anterior (RMS) | 0.046 ± 0.020 | 0.047 ± 0.023 | 0.001 ± 0.004 | 0.040 ± 0.021 | 0.044 ± 0.020 | 0.003 ± 0.003 | 0.846 | **0.006** | **0.006** | 0.084 | 0.076 |
| Gastrocnemius lateralis (RMS) | 0.042 ± 0.017 | 0.044 ± 0.020 | 0.002 ± 0.006 | 0.040 ± 0.019 | 0.045 ± 0.018 | 0.005 ± 0.005 | 0.275 | **0.037** | 0.625 | 0.557 | 0.307 |
| Rectus femoris (RMS) | 0.022 ± 0.012 | 0.022 ± 0.014 | 0.001 ± 0.003 | 0.029 ± 0.016 | 0.033 ± 0.017 | 0.004 ± 0.003 | 0.770 | **0.010** | **0.006** | **0.002** | **0.031** |
| Semitendinosus (RMS) | 0.037 ± 0.015 | 0.037 ± 0.017 | -0.000 ± 0.004 | 0.029 ± 0.012 | 0.031 ± 0.012 | 0.001 ± 0.005 | 0.922 | 0.322 | **0.027** | **0.020** | 0.307 |
